# Multiparametric Magnetic Resonance Imaging and Metabolic Characterization of Patient-Derived Xenograft Models of Clear Cell Renal Cell Carcinoma

**DOI:** 10.3390/metabo12111117

**Published:** 2022-11-15

**Authors:** Joao Piraquive Agudelo, Deepti Upadhyay, Dalin Zhang, Hongjuan Zhao, Rosalie Nolley, Jinny Sun, Shubhangi Agarwal, Robert A. Bok, Daniel B. Vigneron, James D. Brooks, John Kurhanewicz, Donna M. Peehl, Renuka Sriram

**Affiliations:** 1 Department of Radiology and Biomedical Imaging, University of California San Francisco, San Francisco, CA 94143, USA; 2 Department of Urology, Stanford University, Stanford, CA 94305, USA

**Keywords:** renal cell carcinoma, patient-derived xenografts, magnetic resonance imaging, hyperpolarized [1-^13^C]pyruvate, metabolism

## Abstract

Patient-derived xenografts (PDX) are high-fidelity cancer models typically credentialled by genomics, transcriptomics and proteomics. Characterization of metabolic reprogramming, a hallmark of cancer, is less frequent. Dysregulated metabolism is a key feature of clear cell renal cell carcinoma (ccRCC) and authentic preclinical models are needed to evaluate novel imaging and therapeutic approaches targeting metabolism. We characterized 5 PDX from high-grade or metastatic ccRCC by multiparametric magnetic resonance imaging (MRI) and steady state metabolic profiling and flux analysis. Similar to MRI of clinical ccRCC, T_2_-weighted images of orthotopic tumors of most PDX were homogeneous. The increased hyperintense (cystic) areas observed in one PDX mimicked the cystic phenotype typical of some RCC. The negligible hypointense (necrotic) areas of PDX grown under the highly vascularized renal capsule are beneficial for preclinical studies. Mean apparent diffusion coefficient (ADC) values were equivalent to those of ccRCC in human patients. Hyperpolarized (HP) [1-^13^C]pyruvate MRI of PDX showed high glycolytic activity typical of high-grade primary and metastatic ccRCC with considerable intra- and inter-tumoral variability, as has been observed in clinical HP MRI of ccRCC. Comparison of steady state metabolite concentrations and metabolic flux in [U-^13^C]glucose-labeled tumors highlighted the distinctive phenotypes of two PDX with elevated levels of numerous metabolites and increased fractional enrichment of lactate and/or glutamate, capturing the metabolic heterogeneity of glycolysis and the TCA cycle in clinical ccRCC. Culturing PDX cells and reimplanting to generate xenografts (XEN), or passaging PDX in vivo, altered some imaging and metabolic characteristics while transcription remained like that of the original PDX. These findings show that PDX are realistic models of ccRCC for imaging and metabolic studies but that the plasticity of metabolism must be considered when manipulating PDX for preclinical studies.

## 1. Introduction

In 2022 it is estimated that ~79,000 Americans will be diagnosed with kidney cancer and ~14,000 will die from this disease [1]. There has been a consistent increase in the diagnosis of kidney cancer, in part due to incidental imaging findings, with no decline in mortality. Over-diagnosis of insignificant disease and lack of detection of aggressive cancer at an early stage continue to hamper efforts to improve outcomes. Despite the availability of multiple targeted therapies for renal cell carcinoma (RCC), metastatic disease seems inevitable and remains incurable [2].

Authentic preclinical models are needed to improve all aspects of clinical management of kidney cancer from diagnosis to prognosis to treatment. Patient-derived xenografts (PDX) are widely considered the most representative experimental models of human disease [3]. For the most part, PDX retain the genomic, transcriptomic and proteomic characteristics of the tissue of origin with high fidelity and are widely used to investigate cancer biology, therapeutic response and biomarkers predictive of response [4]. Numerous PDX have been established from RCC and, as for other types of cancer, appear to be reliable models of the disease [5].

Minimum information for standardization of PDX has been established and OMICS are a desirable component of validation and quality control [6]. However, while the genome and transcriptome of PDX derived from kidney and other types of cancers have been extensively characterized, less attention has been paid to the metabolome. Dysregulated metabolism, one of the hallmarks of cancer [7], is strongly implicated in the development and progression of clear cell RCC (ccRCC), the most common type of RCC [8]. ccRCC is a broad-spectrum metabolic disease exhibiting dysregulation of oxygen sensing, energy sensing and nutrient sensing signaling cascade pathways [9]. The high frequency of *VHL* gene aberrations in ccRCC leads to stabilization of hypoxia inducible factor (HIF) and exhibition of the classic “Warburg effect”, an elevation in glycolysis in the presence of oxygen [10]. The ubiquitous loss of fructose-1,6-biphosphatase (FBP1) in ccRCC is also key to the Warburg effect [11].

Here, we used several approaches to comprehensively define the metabolic phenotype of five PDX derived from primary and metastatic ccRCC. The metabolism of [U-^13^C]glucose was evaluated by 1D and 2D high-field nuclear magnetic resonance (NMR) spectroscopy. Measurement of steady state levels and fractional enrichment of metabolites was accompanied by ^13^C isotopomer modeling. Multiparametric ^1^H magnetic resonance imaging (MRI), including T_2_ weighted anatomic and diffusion weighted imaging, characterized architectural features of the PDX and hyperpolarized (HP) [1-^13^C]pyruvate evaluated glycolytic activity which is associated with cancer progression and therapeutic response [12]. In addition to studies with ccRCC PDX, cells were cultured from four of the PDX and implanted in mice to establish cell culture-derived xenografts (XEN). The morphologic and metabolic features of XEN were compared to the corresponding PDX to evaluate retention of the phenotype of origin.

## 2. Materials and Methods

### 2.1. Establishment of PDX and XEN

All methods carried out in this study were in accordance with institutional guidelines and regulations. All procedures involving animals and their care were approved by the Institutional Animal Care and Use Committee of Stanford University and the University of California San Francisco in accordance with institutional and National Institutes of Health guidelines. PDX were established from ccRCC tissues obtained from patients undergoing nephrectomy or autopsy between September 2011 and May 2012 at Stanford University under an institutional review board–approved protocol with informed consent as previously described [13,14]. Briefly, tissue cores of tumors were precision-cut into 300 μm thick slices and implanted under the renal capsule of 6–8-week-old RAG2^−/−^γC^−/−^ mice. First-generation tumors were harvested and precision-cut slices were cryopreserved in 95% fetal bovine serum (FBS) and 5% DMSO to establish stock for serial transplantation in mice. PDX at first-generation and at the passage used for imaging and metabolic characterization were characterized by immunohistochemistry (IHC), short-tandem repeat (STR) analysis, and mutational status of the *VHL* gene.

To generate cell culture-derived xenografts, PDX tumors were harvested and minced in HEPES-buffered saline (HBS) into 1–3 mm^3^ fragments. Tissue fragments were washed in HBS once and digested in DMEM/F12 medium supplemented with 10% FBS, 5 μg/mL transferrin, 100 ug/mL gentamycin and 200 U/mL collagenase for 2–4 h at 37 °C on an orbital shaker. The digested tissue was washed once with HBS and passed through a 70 μm cell strainer. The flow-through was spun down and cells were cultured in a T75 flask with DMEM/F12 medium supplemented with 10% FBS, 5 μg/mL transferrin and 100 μg/mL gentamycin and incubated in a humidified incubator at 37 °C with 5% CO_2_. Medium was replaced twice a week. After two passages in vitro, cells were embedded in collagen as previously described before implantation into mice [15]. Briefly, 1 million cells were suspended in 100 μL of type I rat tail collagen at pH 7 and the suspension was dropped onto a cell culture dish and incubated at 37 °C for 20 min to solidify. The collagen plug containing 1 million cells was implanted under the renal capsule of 6–8-week-old RAG2^−/−^γC^−/−^ mice as previously described [15] and xenograft growth was monitored by MRI. The resulting cell culture-derived xenografts (XEN) were characterized by IHC, STR analysis and *VHL* mutation status and precision-cut and cryopreserved for serial transplantation in mice.

### 2.2. Immunohistochemistry (IHC) 

General protocols for IHC of formalin-fixed paraffin-embedded tissues were as previously described [13]. The sources and dilutions of the antibodies used in this study were as follows: Ku70 (Abcam, Cambridge, UK: ab108604 1:200); PAX8 (Sigma, St. Louis, MO, USA 363 M-1 1:200); CAIX (Novus Bio, Centennial, CO, USA: NB100-417 1:500); CD117 (Biocare, Yorba Linda, CA, USA: CM296 1:200); and CD45 (Dako, San Bernardino, CA, USA, M07010 1:200). 

### 2.3. Short-Tandem Repeat (STR) Profiling

Genomic DNA was extracted from tumor tissues preserved in Allprotect tissue reagent (Qiagen, Hilden, Germany) using an AllPrep DNA/RNA/Protein Mini Kit (Qiagen, Hilden, Germany) according to manufacturer’s directions. Twenty microliters of DNA at a 10 ng/µL concentration from each sample were sent to The Genetic Resources Core Facility at The Johns Hopkins University for STR analysis using a GenePrint 10 kit. Profile search was performed against the repositories ATCC and DSMZ. 

### 2.4. VHL Sequencing

Genomic DNA was extracted from tumor tissues preserved in Allprotect tissue reagent using an AllPrep DNA/RNA/Protein Mini Kit according to manufacturer’s directions. The 3 exons of *VHL* were selected for polymerase chain reaction amplification and direct sequencing. The primers used for exon amplification were as follows: Exon 1—Forward: 5’-CTACGGAGGTCGACTCGGGAG-3’; Exon 1—Reverse: 5’-GGGCTTCAGACCGTGCTATCG-3’; Exon 2—Forward: 5’-CCGTGCCCAGCCACCGGTGTG-3’; Exon 2—Reverse: 5’-GGATAACGTGCCTGACATCAG-3’; Exon 3—Forward: 5’-CGTTCCTTGTACTGAGACCCTA-3’ and Exon 3—Reverse: 5’-GAACCAGTCCTGTATCTAGATCAAG-3’.

### 2.5. MRI Protocol

Cryopreserved or fresh tissue slices of PDX or XEN tumors were implanted under the renal capsule of male or female (according to the gender of the original tissue donor) 8–10-week-old NOD.Cg-Prkdcscid Il2rgtm1Wjl/SzJ (NSG) or RAG2^−/−^γC^−/−^ mice from Jackson Laboratory. MRI was performed on a 3T Bruker BioSpin (Bruker, Germany). Mice were imaged in supine position. Tumor growth was monitored by acquiring weekly T_2_-weighted images with a ^1^H quadrature volume coil. ^1^H MRI parameters are listed in Supplementary Table S1. Once tumor volumes reached ~0.8 cc, ^1^H diffusion-weighted images (DWI) and ^13^C HP metabolic images were acquired with a dual tuned ^1^H/^13^C volume coil. Mice were intravenously infused with ~350 mL of 80 mM polarized [1-^13^C]pyruvate over 12 s and 15 HP ^13^C spectra were acquired every 4.2 s beginning 10 s after the start of the injection using a 2D chemical shift imaging pulse sequence with the following parameters: slice thickness of 8 mm, FOV 32 mm × 32 mm, matrix 8 × 8, flip 10°.

### 2.6. MRI Data Analysis

Analysis of MRI data was performed using MATLAB (Mathworks, Natick, MA, USA). Regions of interest (ROI) were manually drawn on T_2_-weighted images covering the contralateral kidney and tumor. In this study, we performed histogram analysis to evaluate intra- and inter-tumoral morphology from T_2_-weighted images. All histograms were plotted in terms of the probability density function (PDF) that displays the relative frequency and probability of a range of values with the AUC being 1. To account for inter-subject variability and enable comparisons among tumors, T_2_-weighted intensity in the whole tumor was normalized by the mean T_2_-weighted intensity of the contralateral kidney for each mouse. These T_2_-weighted normalized intensity values were used for the histogram analysis of tumor morphology, such as the evaluation of the percentage of hypointense (% hypointense) and hyperintense (% hyperintense) in tumors. Pixels with intensities greater than mean + 3 × standard deviation (SD) were considered “hypointense” and those with intensities less than mean—3 × SD were denoted as “hyperintense”. The % hypointense and % hyperintense regions in tumors were calculated as:

(1) $$\%\;hypointense=\frac{number\;of\;hypointense\;pixels\;}{number\;of\;pixel\;in\;the\;whole\;tumor\;}\;\times \;100\%\;$$

(2) $$\%\;hyperintense=\frac{number\;of\;hyperintense\;pixels\;}{number\;of\;pixel\;in\;the\;whole\;tumor\;}\;\times \;100\%\;$$

For ADC analysis, the ROIs in the T_2_-weighted images were downsized to a matrix of 96 × 96 and overlaid on DWI. ADC maps were calculated using the following mono-exponential equation:

(3) $$\frac{S_{b}}{S_{0}}=e^{\left(-b\times ADC\right)}$$

where S_b_ is the signal intensity for each b value, and S_0_ is the signal intensity at a b = 0 s/mm^2^. The ADC mean was calculated for each voxel with a quality of fit (R^2^) higher than 0.7 and ADC values higher than 0.1 × 10^−3^ mm^2^/s for all slices with tumor. 

SIVIC package was used to generate quantitative maps for lactate and pyruvate [16]. Only voxels with signal to noise ratio (SNR) > 3 and more than 5 dynamic time points were considered for quantification purposes. The glycolytic metabolism in tumors was evaluated by calculating the lactate-to-pyruvate area under the curve ratio (L/P AUC ratio). L/P AUC ratio was computed in MATLAB as the area under curves of lactate normalized to pyruvate over time.

### 2.7. Statistical Analysis of Imaging Data 

Data are expressed as mean ± SD. A one-way ANOVA was used to compare differences in T_2_-weighted intensity features, ADC values, L/P AUC ratio, and tumor growth rate among PDX tumors followed by a Tukey’s test for multiple comparisons. Differences between two generations of tumors of the same PDX, or PDX tumors and its corresponding XEN tumors, were assessed with an unpaired t-test. Differences were considered significant at a *p*-Value < 0.05. All statistical analyses were performed with GraphPad Prism 9 (GraphPad Software Inc., San Diego, CA, USA).

### 2.8. ^13^C-Labeling and Extraction of Metabolites

Metabolic labeling of PDX and XEN tumors was achieved by injecting 80 μL of 25% wt/vol [U-^13^C]glucose into the tail vein every 15 min for a total labeling time of 45 min [17]. To minimize the effects of stress and anesthesia on metabolism, mice were briefly anesthetized using isoflurane for 2–3 min to perform ^13^C-injection, then allowed to wake between injections. Tissue was collected immediately upon euthanasia and flash-frozen in liquid nitrogen.

Metabolites were extracted from the frozen tissues using cold 1:1:1 methanol:water:chloroform [18]. Frozen tissues were weighed and thoroughly homogenized in 400 μL of cold methanol at 4 °C using a TissueLyser LT (Qiagen, Hilden, Germany). The aqueous fraction was isolated, lyophilized, and resuspended in 600 μL of D_2_O for NMR analysis. 3-(Trimethylsilyl)propionic-2,2,3,3-d_4_ acid sodium salt (TSP) was added to the aqueous samples to serve as an internal standard for the quantification of metabolites.

### 2.9. NMR Acquisition and Quantification

NMR experiments were performed on an 800 MHz Bruker AvanceI (Bruker, Germany) equipped with a 5 mm triple resonance TXI cryoprobe. High-resolution 1D ^1^H presaturation spectra were acquired with a 90° flip angle, 32 k points, 15 ppm sweep width, 12 s water presaturation, 1.4 s acquisition time, 64 scans, 14 min scan time. ^13^C-decoupled ^1^H water presaturation spectra were acquired with the following parameters: 90° flip angle, 24 k points, 10 ppm sweep width, 12 s water presaturation, 0.5 s acquisition time, 32 scans, 7 min scan time. Adiabatic decoupling was applied during acquisition using a CHIRP pulse with 54 kHz sweep width (equivalent to 200 ppm @ 800 MHz), and shaped pulse power level of +2 dB with respect to the power level determined for ^13^C-GARP. Phase-sensitive 2D ^1^H-^13^C HSQC was used with the following parameters: 2048 × 4096 points, 6 × 120 ppm sweep width, 1.8 s repetition time, 0.297 s acquisition, 2 averages, J_CH_ = 135 Hz (average J_CH_ of 127, 130, and 145 Hz for glutamate C2, C3, and C4), 4 h scan time. ^13^C decoupling was not applied during acquisition to identify ^13^C isotopomers. Data were zero-filled in both dimensions to a final digital resolution of 2.9 Hz/point in the F2 dimension. 2D ^1^H-^1^H TOCSY with water presaturation was used with the following parameters: 4096 × 512 points, 12 × 12 ppm sweep width, 2.2 s repetition time, 0.239 s acquisition time, 8 averages, 60 millisecond mixing time, 2.75 h scan time. Data were zero-filled in both dimensions to a final digital resolution of 9.4 Hertz/point in the F2 dimension.

1D NMR datasets were processed using Mnova 14.0 (Mestrelab Research S.L., Santiago de Compostela, Spain). Each NMR spectrum was zero filled to 65,536, apodised with a 2.0 Hz exponential filter, and manually phased and baseline corrected. Absolute concentrations of 17 steady state metabolites that showed well-resolved resonances were determined from ^13^C-decoupled ^1^H water presaturation 1D spectra of ^13^C-labeled tissue extracts by manual peak picking and fitting of peaks using Mnova 14.0. The relative concentrations to TSP of 11 steady state metabolites that were not well-resolved in 1D spectra were determined using 2D TOCSY cross peaks. 

Fractional enrichment (FE) of key metabolites was quantified using a combination of 1D and 2D NMR methods, where FE = [^13^C-labeled metabolite]_HSQC_/[total metabolite]_{13C}1H_. 2D NMR datasets were zero-padded by a factor of 2, manually phased, and peak volumes were integrated using TopSpin (version 4.0.6) (Bruker, Billerica, MA, USA). The concentration of ^13^C-labeled metabolites was quantified using ^1^H-^13^C HSQC as previously described [19].

### 2.10. ^13^C Isotopomer Modeling

For [U-^13^C]glucose-labeled tumor extracts, relative peak volumes of glutamate C2, glutamate C3 and glutamate C4 isotopomer multiplets were quantified from high-resolution 2D ^1^H-^13^C HSQC spectra using Topspin (version 4.0.6). ^13^C isotopomer modeling of [U-^13^C]glucose-labeled tissues was performed using tcaCALC software (download available at http://invivometabolism.org/tca.html (accessed on 15 September 2020)). This software helps in modeling the active pathways contributing to the carbons in the TCA cycle [i.e., pyruvate dehydrogenase (PDH), pyruvate kinase (PK), pyruvate carboxylase (Y_PC_), and unidirectional anaplerotic flux of substrates leading to succinyl-CoA relative to citrate synthase (Y_S_)]. The isotopomer model was validated using the following metrics: (1) Akaike Information Criterion (AICc), the metabolic model with lowest AICc is most likely to be the best model for the given data set, (2) Ln (Residual), natural logarithms of the sum of squares residual being minimal and (3) ADp, *p*-Value for the Anderson-Darling test for random residuals. Lower ADp (i.e., <0.05) suggests the residuals are not randomly distributed and the model may be overfitted.

### 2.11. Data Analysis of Metabolic Assays

All data are presented as mean ± standard error (SE). All statistical analyses were performed using Prism (version 8.3, GraphPad Software, SanDiego, CA, USA). The statistical significance in the steady state concentration of metabolites among the PDX or XEN was assessed by two-way ANOVA. Multiple statistical comparisons of the lactate FE and glutamate FE among the PDX or XEN were calculated using ordinary one-way ANOVA with ‘Tukey’ post hoc analysis. Multiple *t*-test was used to compare the concentration of metabolites between the PDX and their corresponding XEN. Statistical significance of lactate FE and glutamate FE between the PDX and their corresponding XEN was assessed by an unequal variance (Welch’s) *t*-test. *p*-Values less than 0.05 (* *p* < 0.05, ** *p* < 0.01 and *** *p* < 0.001) were considered significant.

### 2.12. RNA-Sequencing (RNA-Seq) and Analysis

Total RNA was isolated from flash-frozen tissues using an AllPrep DNA/RNA mini kit (Qiagen, Hilden, Germany) and integrity was confirmed using an Agilent Bioanalyzer (Agilent Technologies, Santa Clara, CA, USA). RNA libraries were prepared using the PrepX mRNA library kit (Wafergen Bio-Systems, Fremont, CA, USA). RNA sequencing was carried out by Novogene Corporation Inc. (Sacramento, CA, USA) on the Illumina Hiseq system with paired-end reads up to 150 bp. Sequencing reads were aligned to the Gencode (GRCh38.p36) reference genome using STAR version 2.5.4b [20] (http://code.google.com/p/rna-star/, accessed on 20 March 2018) and gene counts produced with HTSeq [21]. Transcript counts were normalized to the total number of transcripts detected in each sample. The data were then filtered for genes with an average expression, taken across all samples, greater than one transcript per million. Gene expression data have been deposited in Gene Expression Omnibus (GEO Accession: GSE213857). The R packages ComplexHeatmap, Circlize, Vegan, ggplot2, and Corrplot were used for clustering analysis, principal component analysis and matrix correlation analysis with the PDX and XEN RNA-seq data (R software 4.2.1 https://www.r-project.org (accessed on 24 June 2022)). Pearson correlation analysis with the mean value of each PDX and XEN tumor was performed with Prism (version 9, GraphPad Software, SanDiego, CA, USA).

## 3. Results

### 3.1. Genetic and Immunohistologic Characterization of ccRCC PDX and Cell Culture-Derived Orthotopic Xenografts

Five PDX established from high grade primary or metastatic ccRCC specimens were used in this study. The clinicopathological characteristics of the donors and tumors from which the PDX were derived are listed in Supplementary Table S2. In addition, cells were cultured from four of the PDX and implanted into mice to generate cell culture-derived xenografts (XEN). Representative tumors from each PDX and XEN were characterized for expression of a panel of biomarkers by immunohistochemistry (IHC) (Supplementary Figures S1 and S2). Hematoxylin- and eosin-stained tissues of all PDX and XEN showed histomorphologic characteristics of ccRCC. Positive staining by antibody against Ku70 demonstrated the human origin of each tumor and negative staining for CD45 ruled out origin from EBV-transformed lymphoblasts. All PDX and XEN were positive for CAIX and PAX8, classic biomarkers of ccRCC, and negative for CD117, a biomarker of papillary RCC. Short-tandem repeat (STR) profiling confirmed the independent identity of each PDX and the STR profile of each XEN matched that of the PDX from which it was derived (Supplementary Table S3). Exon sequencing of the VHL gene revealed mutations in 4 of the 5 PDX, with retention of the VHL genotype in the corresponding XEN (Supplementary Table S4). Overall, the 5 PDX and 4 XEN exhibited genetic and immunopathologic features characteristic of ccRCC. 

### 3.2. Comparison of Multiparametric MRI Morphologic Features among PDX 

**Figure 1:**
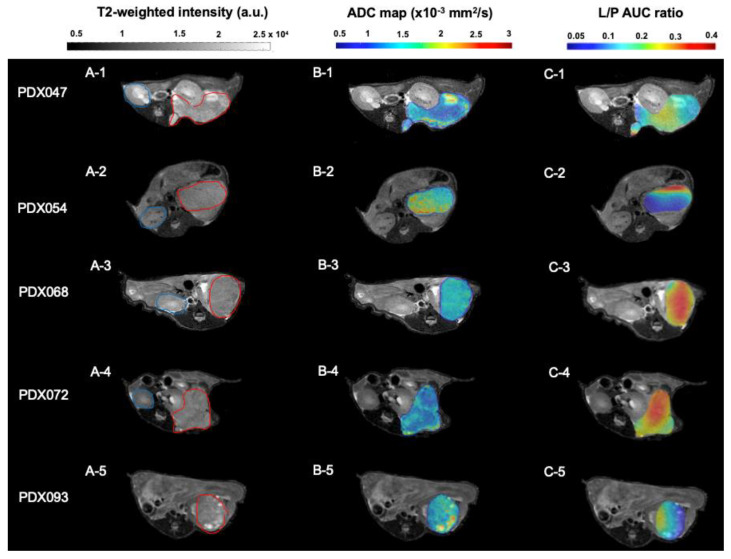
(**A1**–**A5**) Representative T2-weighted MR images of PDX implanted under the murine renal capsule. Blue and red lines were drawn along the borders of the contralateral kidney and tumor, respectively. Images show intra-tumoral heterogeneity in some PDX, especially hyperintense components. (**B1**–**B5**) Representative ADC maps overlaid on the T2-weighted images of PDX tumors. ADC images highlight differences in water diffusion among PDX with intra-tumoral heterogeneity in most tumors. (**C1**–**C5**) Representative L/P AUC ratio maps overlaid on the T2-weighted images of PDX tumors, reflecting intra-tumoral variation in glycolytic metabolism in all tumors. a.u.= arbitrary units; AUC = area under the curve.

**Figure 2:**
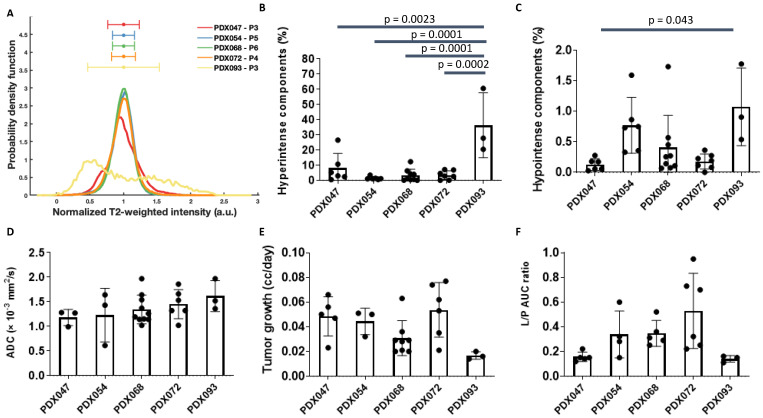
Multiparametric MRI of PDX tumors. (**A**) The normalized overlay (average representation of all tumors of each PDX) of T2-weighted intensities. Bar graphs show the % hyperintense (**B**) and % hypointense (**C**) components in all PDX tumors calculated from histograms of T2-weighted images. (**D**) ADC mean calculated for all PDX tumors showed similar water diffusion among PDX. (**E**) The tumor growth rate (cc/day) was calculated as the slope of the last 3 tumor volumes prior to HP MRI and the time (in days) between the first and final measurement. (**F**) The mean of the L/P AUC ratios for all PDX tumors. The significance of the one-way ANOVA with a Tukey’s multiple comparisons test is indicated above the plots. All *p* < 0.05 indicated statistical significance. a.u.= arbitrary units; AUC = area under the curve.

Each PDX was implanted under the renal capsule of gender-appropriate mice (according to the gender of the respective tissue donor). Imaging characterization was performed on tumor volumes ranging from 0.15–1.0 cc. T_2_-weighted images of representative tumors from each of the 5 PDX are shown in Figure 1A. Except for PDX093, T_2_-weighted intensities of most of the tumors from a given PDX (3 to 9 tumors per PDX) were normally distributed (Figure 2A and Figure S3). Tumors from PDX047, PDX054, PDX068 and PDX072 were homogeneous, with little variation in signal intensity within each of the tumors. In contrast, tumors from PDX093 exhibited considerable intra-tumoral heterogeneity, with some obvious hyperintense regions. As detailed in the methods section, a threshold of 3 standard deviations (SD) from the normalized mean was used to determine % hyperintense (+3 SD) and % hypointense (−3 SD) components, related to cystic and necrotic regions, respectively. The % hyperintense components among tumors of the different PDX ranged from the least in PDX054 (1.38 ± 1.05%) to the highest (36.25 ± 21.36%) in PDX093, with the other PDX tumors exhibiting <10% hyperintense regions (Figure 2B). The significantly larger % hyperintense components in PDX093 compared to all other PDX suggested that this PDX was more cystic. The % hypointense components were overall negligible, ranging from 0.13 ± 0.10% in PDX047 to 1.07 ± 0.63% in PDX093, suggesting little necrosis in these tumors (Figure 2C). 

ADC maps overlaid on T_2_-weighted images of tumors representative of each PDX are shown in Figure 1B. The mean ADC for PDX tumors ranged from 1.178 ± 0.176 × 10^−3^ mm^2^/s (PDX047) to 1.615 ± 0.321 × 10^−3^ mm^2^/s (PDX093) but was not significantly different among the 5 PDX (Figure 2D and Figure S4). Tumor growth rates were variable among the PDX and ranged from a mean of 0.017 cc/day (PDX093) to 0.053 cc/day (PDX072) but were not significantly different among the PDX (Figure 2E). 

These findings demonstrate that the ccRCC PDX capture the breadth of clinical disease including presence of cysts in some PDX, heterogenous tumor growth rates and similarity of ADC values. The lack of necrotic components of these orthotopic PDX make them optimal for preclinical metabolic imaging studies.

### 3.3. HP [1-^13^C]pyruvate MRI of PDX

Representative maps of the conversion of HP [1-^13^C]pyruvate to lactate (L/P AUC ratio) derived from dynamic HP ^13^C MRI data were overlaid on T_2_-weighted images (Figure 1C). L/P AUC ratios show inter-PDX differences in glycolytic metabolism (Figure 2F). PDX072 tumors were highly glycolytic (mean L/P AUC ratio 0.52 ± 0.30) while PDX093 tumors, which had the highest % of cystic and necrotic components (Figure 2B,C) and the lowest tumor growth rate (Figure 2E), exhibited the lowest glycolysis (mean L/P AUC ratio 0.14 ± 0.02). However, differences in L/P AUC ratio did not reach statistical significance among PDX. Furthermore, the intra-PDX differences in PDX047 and PDX093 were minimal relative to the other PDX. We have thus demonstrated the feasibility of metabolic imaging using HP ^13^C MRI in ccRCC PDX and observe that the intra-PDX variance is higher in tumors with higher glycolytic capacity.

### 3.4. Comparison of Imaging Features of Two Different Passages of a PDX

Tumor morphology (from T_2_-weighted images), cellularity (based on ADC values) and glycolytic metabolism (L/P AUC ratios) were compared between passage 3 (P3) and P4 of PDX047 (Supplementary Figure S5A–C). While T_2_-weighted intensities of P4 tumors were not significantly different relative to P3, the values demonstrated much larger (10-fold) inter-passage variation in the hyperintense pixel composition (cystic regions) (Supplementary Figure S5D). For both passages, necrotic regions were negligible (Supplementary Figure S5E). PDX047 P3 exhibited significantly lower ADC values compared to PDX047 P4 (P3: 1.18 ± 0.16 × 10^−3^ mm^2^/s vs. P4: 1.74 ± 0.10 × 10^−3^ mm^2^/s, *p* = 0.02), suggesting lower tumor cellularity in the later passage (Supplementary Figure S5F). Interestingly, P4 tumors showed >3-fold higher glycolytic metabolism (L/P AUC ratio) relative to P3 (*p* = 0.003) (Supplementary Figure S5G). Tumor growth rate was not different between P3 and P4 (Supplementary Figure S5H). These findings, although limited in nature, suggest that passage of ccRCC PDX may result in more homogeneous and glycolytic tumors with lower cellularity and less cystic development.

### 3.5. Comparison of Imaging Features between PDX and XEN 

**Figure 3:**
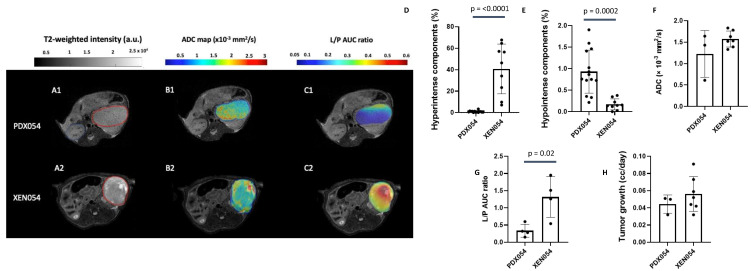
Comparison of features of PDX054 and XEN054. Panel on left: (**A1**,**A2**) Representative T2-weighted MR images of PDX and XEN implanted under the murine renal capsule. Red lines were drawn along the borders of the tumors. (**B1**,**B2**) Representative ADC maps overlaid on the T2-weighted images of PDX and XEN tumors. (**C1**,**C2**) Representative L/P AUC ratio maps overlaid on the T2-weighted images of PDX and XEN tumors. A.u.= arbitrary units; AUC = area under the curve. Bar graphs show: the % hyperintense (**D**) and % hypointense (**E**) components in all PDX and XEN tumors calculated from histograms of T2-weighted images; (**F**) ADC mean calculated for all PDX and XEN tumors; (**G**) the mean of the L/P AUC ratios for all PDX and XEN tumors; and (**H**) the tumor growth rate (cc/day). The significance of the one-way ANOVA with a Tukey’s multiple comparisons test is indicated above the plots. All *p* < 0.05 indicated statistical significance.

Cell cultures were established from PDX047, PDX054, PDX072 and PDX093. After expansion in vitro, cells were implanted under the renal capsule of mice to generate xenografts (XEN047, XEN054, XEN072 and XEN093). Imaging features of 3 of the 4 XEN (XEN047 was not imaged) were compared to their PDX of origin. Representative multiparametric MRI features (T_2_-weighted intensity, ADC map and L/P AUC ratio) of PDX054 and XEN054 are shown in Figure 3A–C. Overall, XEN054 showed 40% significantly higher hyperintense regions, associated with cystic components, compared to PDX054 (*p* = 0.0001) (Figure 3D). Although both PDX054 and XEN054 tumors showed very low % hypointense regions, the percentage was higher for the PDX tumors (*p* = 0.0002) (Figure 3E). While ADC values for PDX054 and XEN054 were not statistically different (Figure 3F), XEN054 tumors showed >3-fold higher glycolytic metabolism (L/P AUC ratio) compared to PDX054 tumors (*p* = 0.02, Figure 3G). There was no significant difference in tumor growth rate between PDX and XEN tumors (Figure 3H). For PDX054, passage of tumor cells through culture and back into mice appeared to change the phenotype of the resulting XEN tumors in several ways, including increasing glycolysis and morphological changes with higher cystic components.

**Figure 4:**
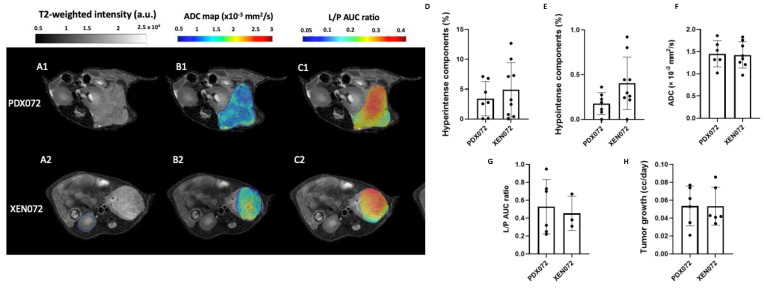
Comparison of features of PDX072 and XEN072. Panel on left: (**A1**,**A2**) Representative T2-weighted MR images of PDX and XEN implanted under the murine renal capsule. Red lines were drawn along the borders of the tumors. (**B1**,**B2**) Representative ADC maps overlaid on the T2-weighted images of PDX and XEN tumors. (**C1**,**C2**) Representative L/P AUC ratio maps overlaid on the T2-weighted images of PDX and XEN tumors. a.u.= arbitrary units; AUC = area under the curve. Bar graphs show: the % hyperintense (**D**) and % hypointense (**E**) components in all PDX and XEN tumors calculated from histograms of T2-weighted images; (**F**) ADC mean calculated for all PDX and XEN tumors; (**G**) the mean of the L/P AUC ratios for all PDX and XEN tumors; and (**H**) the tumor growth rate (cc/day). The significance of the one-way ANOVA with a Tukey’s multiple comparisons test is indicated above the plots. All *p* < 0.05 indicated statistical significance.

Figure 4A–C shows representative multiparametric MRI comparisons between PDX072 and XEN072. In contrast to the previously described PDX054/XEN054 models, PDX072 and XEN072 tumors exhibited similar T_2_-weighted intensities (Figure 4A), with less than 5% hyperintense regions (Figure 4D) and a negligible percentage of hypointense regions (less than 0.5%) (Figure 4E). Moreover, PDX072 and XEN072 tumors showed a similar mean ADC among the tumors (~1.4 × 10^−3^ mm^2^/s) (*p* = 0.87) (Figure 4F). There were also no significant differences in L/P AUC ratios (Figure 4G) or tumor growth rate (Figure 4H) between PDX072 and XEN072. In contrast to PDX/XEN054, passage of PDX072 cells through culture and back into mice did not appear to appreciably alter the phenotype of the resulting XEN tumors.

The phenotypes of PDX093 and XEN093 were relatively similar. Representative T_2_-weighted intensities, ADC maps and L/P AUC ratios of tumors are shown in Supplementary Figure S6A–C. Mean T_2_-weighted intensities, % hyperintense regions, ADC and growth rates were not different between PDX093 and XEN093 (Supplementary Figure S6A,D,F,H). The most significant difference between PDX093 and XEN093 was in the negligible necrosis (% hypointense regions) in XEN compared to PDX tumors and an ~2-fold increase in glycolysis (L/P AUC ratio) in the XEN tumors (Supplementary Figure S6E,G). These significant changes were like those seen in the PDX/XEN054 pair, suggesting that these may be common alterations induced by passage of tumor cells through culture.

In brief, there was no specific change that was consistently observed between the PDX and XEN pairs in morphology, functional or metabolic parameters, pointing to the variable influence of the in vitro culture on the heterogeneous PDX. 

### 3.6. Metabolic Profiling and Flux Analyses of PDX and XEN 

**Figure 5:**
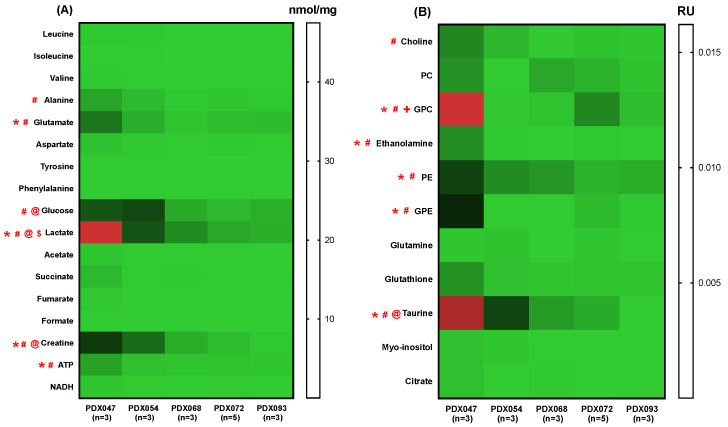
Heat maps showing (**A**) absolute concentrations (nmol/mg) and (**B**) concentrations relative to TSP (RU) of steady state metabolite levels among PDX. *: *p* < 0.05, PDX047 and PDX054, #: *p* < 0.05, PDX047 and other PDX except PDX054, @: *p* < 0.05, PDX054 and other PDX except PDX047, $: *p* < 0.05, between PDX068 and PDX072, and PDX068 and PDX093, +: *p* < 0.05, between PDX072 and PDX093.

PDX and XEN tumors were harvested from mice infused with [U-^13^C]glucose. Soluble metabolites were extracted and identified by high-field NMR. The heat maps in Figure 5A,B show absolute and relative concentrations of steady state metabolites, respectively, among PDX. Steady state levels of glucose, lactate, alanine, glutamate, creatine, adenosine triphosphate (ATP), taurine, choline, glycerophosphocholine (GPC), ethanolamine, phosphoethanolamine (PE) and glycerophosphoethanolamine (GPE) were significantly different among the PDX. PDX047 and PDX054 were distinctive with the greatest number of significantly different levels of metabolites compared to the other three PDX, which were similar to each other. The distinguishing metabolites were typically elevated in PDX047 and then PDX054 followed by a gradual decrease going from PDX068 to PDX093. Further, comparison of the steady state metabolic profiles of P3 and P4 of PDX047 indicated variability in the metabolic features of PDX of different passages (Supplementary Figure S7A,B). The levels of several metabolites that differed among PDX (glutamate, glucose, lactate, creatine, GPC, GPE and taurine) were significantly lower in P4 compared to P3. 

Metabolite levels were relatively similar among the four XEN, although significantly different levels of glutamate, glucose, lactate, creatine, GPC, PE, GPE and taurine were noted between some of the XEN (Supplementary Figure S8A,B), with XEN047 and XEN054 having the highest levels (with the exception of XEN072 for GPC similar to that of PDX072). However, of note is that the trend of decreasing lactate levels observed when going from PDX047 to PDX093 is not recapitulated in XEN tumors. The lactate levels were similar in the three XEN (047, 054 and 072) with XEN 093 alone being significantly lower. 

**Figure 6:**
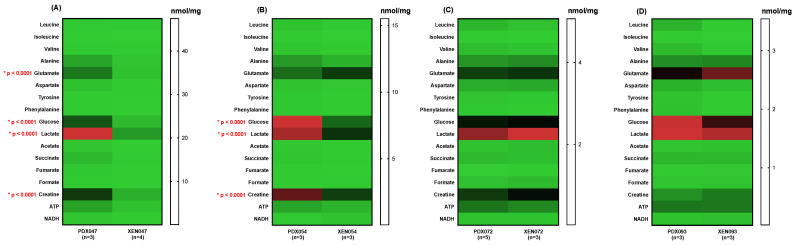
Heat maps showing absolute concentrations of steady state metabolites between PDX and their corresponding cell culture-derived XEN: (**A**) PDX/XEN047; (**B**) PDX/XEN054; (**C**) PDX/XEN072; (**D**) PDX/XEN093. * indicates statistical significance.

**Figure 7:**
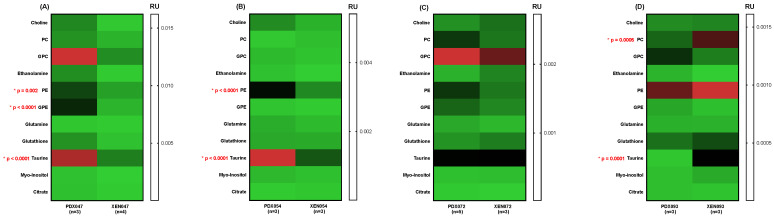
Heat maps of relative concentration (RU) to TSP of steady state metabolites between PDX and their corresponding cell culture-derived XEN: (**A**) PDX/XEN047; (**B**) PDX/XEN054; (**C**) PDX/XEN072; (**D**) PDX/XEN093. * indicates statistical significance.

A comparison of metabolic profiles between PDX and their corresponding XEN showed significant differences between PDX047/XEN047 (Figure 6A and Figure 7A) and PDX054/XEN054 (Figure 6B and Figure 7B). Steady state levels of glucose, lactate, creatine, PE and taurine were significantly higher in PDX047 and PDX054 compared to their corresponding XEN, with higher levels also of glutamate and GPE in PDX047 compared to XEN047. In contrast, steady state metabolite levels were similar between PDX072/XEN072 (Figure 6C and Figure 7C) and between PDX093/XEN093, with only PC and taurine higher in XEN093 compared to PDX093 (Figure 6D and Figure 7D).

**Figure 8:**
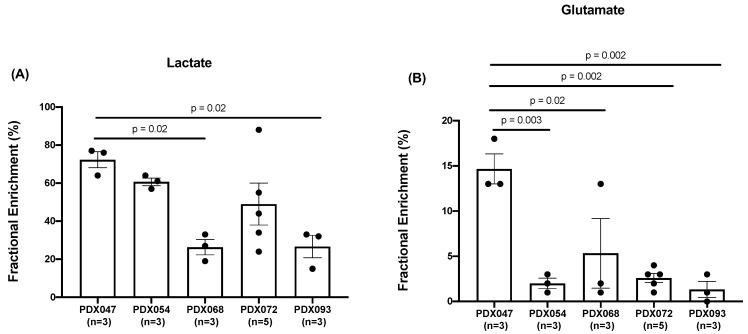
Comparison of fractional enrichment of (**A**) lactate and (**B**) glutamate among PDX.

The fractional enrichment (FE) of lactate and glutamate in [U-^13^C]glucose-labeling studies was quantified in PDX and XEN. PDX047 had significantly higher lactate FE compared to PDX068 and PDX093 (Figure 8A) and significantly increased FE of glutamate compared to all the other PDX (Figure 8B), while there was no significant difference in lactate and glutamate FE amongst the XEN (Supplementary Figure S9). Interestingly, the glucose levels observed in the PDX correlated significantly with the lactate levels as well as the lactate FE in the PDX (Supplementary Figure S10).

**Figure 9:**
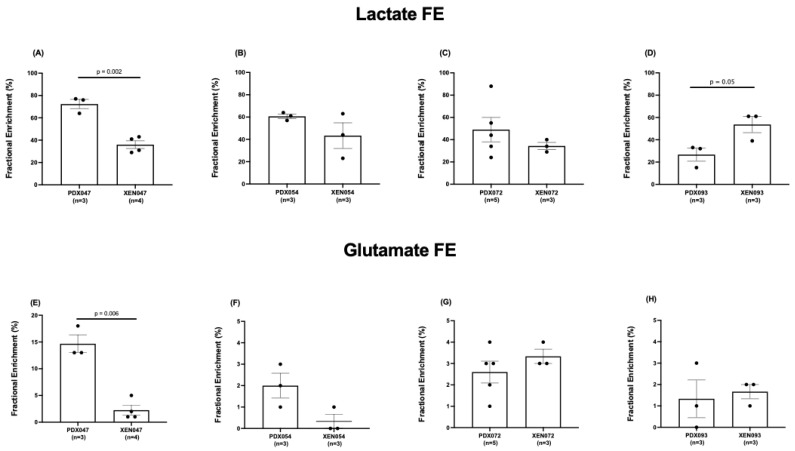
Comparison of fractional enrichment of (**A**–**D**) lactate and (**E**–**H**) glutamate between PDX and corresponding XEN. Statistical test used: unpaired *t*-test with Welch’s correction.

Considering the significant labeling of glutamate pools in PDX047, we analyzed the ^13^C isotopomer data using tcaCALC software to determine if there was any anaplerotic flux of carbons into the TCA cycle and found that the pyruvate carboxylase (PC) pathway contributed to the carbons of the TCA cycle equivalently to pyruvate dehydrogenase (PDH) (Supplementary Figure S11). Comparing different passages of PDX047, FE of both lactate and glutamate was higher in P3 than in P4 (Supplementary Figure S12). Further, comparison of lactate and glutamate FE between the PDX and their corresponding XEN showed that lactate FE was significantly higher in PDX047 compared to XEN047, but lower in PDX093 compared to XEN093, and equivalent between PDX/XEN054 and PDX/XEN072 (Figure 9A–D). Glutamate FE was similar between all PDX and XEN pairs except for PDX/XEN047, in which case glutamate FE was significantly lower in XEN047 (Figure 9E–H).

In summary, the varied range of metabolite and flux levels observed between PDX was blunted by the in vitro culturing process and resulted in more homogenous metabolic and flux profiles in the XEN.

### 3.7. Gene Expression Profiling of PDX and XEN

**Figure 10:**
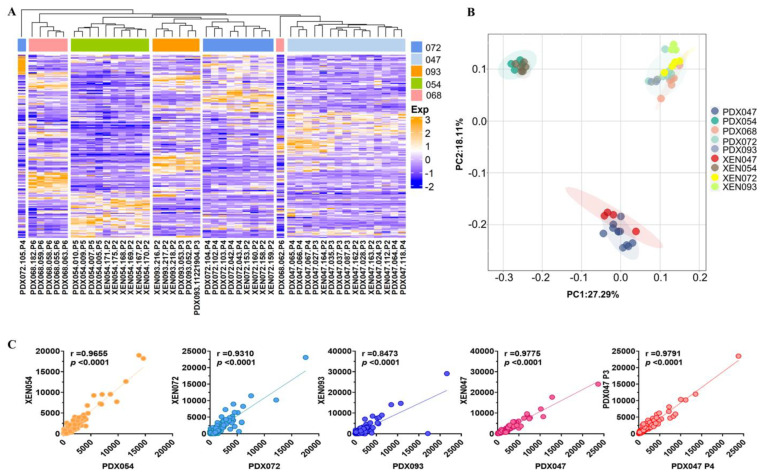
(**A**) The RNA-seq gene expression data from 30 PDX and 17 XEN were subjected to hierarchical clustering analysis and (**B**) principal component analysis with the R Package. (**C**) Pearson correlation analysis between each pair of PDX and cell culture-derived XEN and PDX047 P3 and P4.

RNA-sequencing (RNA-Seq) of PDX and XEN tumors was performed to compare the transcriptomes of the 5 PDX and to evaluate relative retention of the transcriptomes in XEN. Hierarchical clustering analysis based on global gene expression showed that XEN tumors mostly clustered with their PDX of origin (Figure 10A), which was confirmed by principal component analysis (PCA) (Figure 10B). It is interesting to note that the PDX and XEN that showed the most distinctive metabolic phenotypes (PDX/XEN047 and PDX/XEN054) were widely separated from each other and from the other PDX/XEN in the PCA plot, while the PDX/XEN most like each other metabolically (PDX068, PDX/XEN072 and PDX/XEN093) clustered together (Figure 10B). Transcriptomes of each PDX/XEN pair, as well as of PDX047 P3 and P4, were highly correlated (Figure 10C), showing that global gene expression was not dramatically altered by passage of cells in vitro before establishment of XEN or by passage of PDX in vivo. Furthermore, correlation analysis of the glycolysis pathway to MRI features showed significant correlation between glucose levels and GLUT1 transporter (Supplementary Figure S10) and LDHA/LDHB ratio to L/P AUC ratio (Supplementary Figure S13). These data clearly demonstrate the preservation of the transcriptome between passages and between PDX and their respective XEN counterparts. 

## 4. Discussion

To our knowledge, this study represents the first in depth imaging and metabolic characterization of ccRCC PDX. Multiparametric MRI of orthotopic tumors revealed similarities as well as some distinctive differences in imaging features among the 5 PDX. Similar to the mostly homogenous and hyperintense appearance of clinical ccRCC relative to normal renal cortex in T_2_-weighted images [22], tumors from 4 of the PDX showed very homogeneous T_2_-weighted signal intensity, with little intra- or inter-tumoral variation among multiple tumors of a given PDX. In contrast, PDX093 exhibited considerable signal heterogeneity among and within tumors, with hyperintense components significantly higher in this PDX compared to the others. Hyperintense T_2_-weighted signal is associated with cystic development, suggesting that some ccRCC PDX are more prone to cystic formation than others, as is the case for some clinical RCC [23]. The % hypointense areas of tumors, although variable, was <2% in all tumors, suggesting that necrosis was not a predominant feature of these ccRCC PDX. The absence of necrosis, a feature that complicates preclinical studies, is a beneficial effect of propagating PDX under the highly vascularized renal capsule. ADC, influenced by multiple factors including cellularity, cell membrane integrity, nuclear to cytoplasmic ration and viscosity [24], ranged from 0.61 to 1.97 × 10^−3^ mm^2^/sec across PDX tumors, but mean ADC values were not significantly different among the 5 PDX. In a recent clinical study, ADC values < 1.9505 × 10^−3^ mm^2^/sec indicated that lesions were more likely to be RCC or angiomyolipoma than oncocytoma [25], and the ADC values of the PDX were consistent with the clinical findings. Finally, functional imaging by HP [1-^13^C]pyruvate MRI was used to evaluate glycolysis. Consistent with the impact of VHL gene aberrations in ccRCC PDX [10], all PDX demonstrated high mean L/P AUC ratios (high levels of glycolysis). The mean L/P AUC ratio had a wide range among the individual tumors, from 0.11 to 0.95, but was not significantly different among the PDX. Notably, values for L/P AUC ratio were tightly clustered for tumors of some PDX, while tumors of other PDX had highly variable values. A similar phenomenon was observed in a HP [1-^13^C]pyruvate MRI study of colorectal and breast cancer xenografts [26]. The first-in-human HP [1-^13^C]pyruvate MRI of RCC was reported in 2019 [27]. Like genomic heterogeneity of RCC [28], intra-tumoral metabolic heterogeneity was striking. Results from a second patient study suggested that HP [1-^13^C]pyruvate MRI may potentially discriminate low- from high-grade ccRCC [29]. In a recent study of 9 patients with various types of renal tumors, increasing k_PL_ (the apparent exchange rate constant between ^13^C-pyruvate and ^13^C-lactate) correlated with increasing overall tumor grade of ccRCC and expression of MCT1 [30]. The highly glycolytic nature of our 5 PDX is consistent with these clinical observations since these PDX originated from high-grade primary tumors and metastases. 

Comparing multiparametric MRI features of XEN derived by short-term culture of cells from PDX and re-implantation in mice to the PDX of origin showed similarities but also some changes. Overall, XEN tumors generally grew at the same rate as the parental PDX tumors, with growth rates per tumor ranging from 0.013 to 0.127 cc/day. One PDX-XEN pair (072) had identical MRI features, whereas XEN093 showed a significant increase in glycolysis (L/P AUC ratio increased ~2-fold) and an increased hypointense signal component in T_2_-weighted images compared to PDX093. XEN054 showed a striking increase in L/P AUC ratio and % hyperintense areas, and decreased hypointense area, relative to the PDX. While no given feature was consistently different between XEN and PDX pairs, these observations suggest that passage through culture may permanently alter some elements of the phenotype, despite returning the cells to the in vivo microenvironment. Indeed, imaging features may be unstable during in vivo propagation as well. Our exploratory study of two serial passages of PDX047 (P3 and P4) suggested that passage of ccRCC PDX may result in more glycolytic and less cellular tumors. While immunohistologic and genetic studies often show stability of PDX with passage, our findings suggest that morphologic and metabolic phenotypes may not be as stable. Indeed, hierarchical clustering and PCA of global gene expression showed that PDX and XEN tumors tended to cluster together, and similarity was confirmed by high correlation coefficients between gene expression in each PDX and XEN pair and between P3 and P4 of PDX047. 

Metabolic profiling and flux analyses provided deeper insights into the characteristics of ccRCC PDX and XEN. Quantification of steady state levels of metabolites extracted from PDX labeled with [U-^13^C]glucose showed a high degree of similarity among 3 of the PDX (068, 072 and 093). In contrast, the other two PDX (047 and 054) were significantly different from the others. The distinguishing metabolites were all higher in PDX047 and PDX054 and included alanine, glutamate, glucose, lactate, creatine, ATP, Kennedy pathway metabolites (choline, GPC, ethanolamine, PE, GPE) and taurine. Steady state levels of metabolites in XEN derived from PDX072 and PDX093 were remarkably like those in the parental PDX, with higher levels of PC and taurine the only significant differences in XEN093 compared to PDX093. In contrast, levels of numerous metabolites differed between XEN047 and XEN054 compared to their parental PDX. All significantly different metabolites were higher in the PDX compared to XEN (glutamate, glucose, lactate, creatine, PE, GPE, and taurine). For the most part, these same metabolites were higher in XEN047 or XEN054 compared to the other XEN (072 or 093). In this regard, although XEN047 and XEN054 had lower levels of several key metabolites than their parental PDX, they still retained higher levels of metabolites than the other XEN derived from PDX with lower levels of metabolites overall. 

Comparing fractional enrichment (FE) of lactate among the PDX again revealed distinctive differences with FE significantly higher in PDX047 compared to some of the other PDX. Lactate FE in XEN054 and XEN072 was like that in the parental PDX, while it decreased in XEN047 and increased in XEN093 compared to the parental PDX. FE of glutamate was relatively low among the PDX, except for significantly higher FE in PDX047 compared to the other PDX. Glutamate FE significantly decreased in the XEN derived from PDX047, dropping to the low levels (<5% enrichment) characteristic of the other PDX and XEN. There was no apparent labeling of the aspartate, succinate or glutamine pools in the PDX, reinforcing the literature of reduced contribution of glucose-derived carbon to the TCA cycle via both PDH and PC in ccRCC [31]. It is important to note that the FE measured by isotopomer labeling reflects equilibrium kinetics convolved with label losses (biochemical efflux) while the L/P AUC ratio is a function of many factors such as transport of pyruvate into the cell, steady state pool size of the product, co-factor availability as well as the enzyme levels that cannot be disentangled in the in vivo situation due to drastic difference of the time scale of measurement (30 s as opposed to 45 min). 

Analysis of the ^13^C isotopomer data from PDX047 using tcaCALC software suggested that anaplerotic flux of carbons into the TCA cycle was contributed equally by both the PC and PDH pathways, resulting in significant labeling of glutamate pools in PDX047. Taken together with the differences in levels of steady state metabolites and metabolic flux, these results again emphasize that PDX047 and PDX054 are metabolically different from the other three PDX. Notably, tumors arising from PDX047 and PDX054 tended to cluster away from the other tumors in PCA of gene expression, reinforcing the distinction observed in the metabolomics studies of these two PDX. However, XEN derived from these two PDX became like the other XEN and exhibited a simplified glycolysis heavy metabolic profile, suggesting that passage through cell culture altered the TCA cycle. 

Preclinical studies will continue to inform translation of HP ^13^C MRI and other types of metabolic imaging to the clinic. The value of information provided by multi-platform imaging of animal models was recently reviewed by Serkova et al. [32]. In our study, we obtained information about anatomy and physiology of ccRCC PDX and XEN by proton MRI, glycolytic metabolism by HP [1-^13^C]pyruvate MRI, and global metabolism by steady state metabolic profiling and flux analysis. We found that imaging and metabolic features of ccRCC PDX are representative of clinical ccRCC, suggesting that PDX will be appropriate models for preclinical studies targeting metabolism for therapy and/or imaging. However, manipulation of PDX, such as by in vitro cell culture and implantation back into mice as xenografts, may alter the metabolic and imaging phenotype. A limitation of our study was the relatively small number of PDX, XEN-PDX pairs, and PDX passages. Further, relatively small numbers of tumors from each PDX or XEN underwent HP MRI, underpowering multivariate comparisons. Nevertheless, it is interesting to compare our results to similar studies of other PDX. Jun et al. compared levels of metabolites in several pancreatic cancer PDX to the patient tumors of origin and found similar patterns, although the site of propagation of PDX (orthotopic versus subcutaneous) influenced metabolite levels [33]. A study of PDX derived from colorectal cancer showed that metabolic signatures were retained throughout four passages, despite the replacement of human by murine stroma by passage 2 [34]. Comprehensive imaging and metabolic studies of additional types of PDX in the future will contribute to evaluation of PDX as predictive models of human cancer. 

## 5. Conclusions

The metabolic comparisons of the PDX are congruent with the genetic clustering observed among them. However, the genetic stability observed between passages or upon establishment of XEN from cultured cells does not carry over to the metabolomic or imaging features, highlighting the plasticity of the metabolic phenotype and its dependency on microenvironment and growth conditions. These results emphasize the need to understand the irreversible streamlining of metabolic changes conferred upon in vitro culture of cells from PDX and optimal choice of model systems for translational research in RCC including drug screening and biomarker discovery targeting metabolic pathways. 

## Data Availability

The data presented in this study are available in this article and supplementary material.

## Supplementary Materials

The following supporting information can be downloaded at: https://www.mdpi.com/article/10.3390/metabo12111117/s1, Figure S1. Expression of biomarkers by ccRCC PDX; Figure S2. Expression of biomarkers by cell culture-derived XEN; Figure S3. Histograms of normalized T_2_-weighted intensities of all tumors of each PDX (RCCxxx indicates tumor ID); Figure S4. Histograms of ADC of all tumors of each PDX (RCCxxx indicates tumor ID and P is passage); Figure S5. Representative T_2_-weighted MR images (A1,A2), ADC maps (B1,B2) and L/P AUC ratio (C1,C2) for passage 3 (P3) and P4 of PDX047; Figure S6**.** Comparison of features of PDX093 and XEN093; Figure S7. Heat maps showing (A) absolute concentrations and (B) relative concentration (RU) to TSP of steady state metabolite levels between passage 3 (P3) and passage 4 (P4) of PDX047; Figure S8. Heat maps of (A) absolute concentrations and (B) relative concentration (RU) to TSP of steady state metabolite levels among cell culture-derived xenografts (XEN); Figure S9. Comparison of fractional enrichment of lactate and glutamate among cell culture-derived xenografts (XEN); Figure S10. Correlation of metabolite and mRNA levels; Figure S11. ^13^C isotopomer modeling of [U-^13^C]glucose-labeled PDX047 using tcaCALC software; Figure S12. Comparison of fractional enrichment of (A) lactate and (B) glutamate between passage 3 (P3) and passage 4 (P4) of PDX047; Figure S13. Correlation of relevant mRNA levels to L/P AUC ratio; Table S1. ^1^H MR imaging parameters; Table S2. Clinicopathologic characteristics of ccRCC specimens; Table S3. STR profiles of PDX and XEN; Table S4. VHL genotype of PDX and XEN.
