# Supplementary material for: Multiparametric Magnetic Resonance Imaging and Metabolic Characterization of Patient-Derived Xenograft Models of Clear Cell Renal Cell Carcinoma
Source: Metabolites. 2022 Nov 15;12(11):1117. doi: 10.3390/metabo12111117 (PMC9692472; doi:10.3390/metabo12111117)
Supplement: Supplementary file 1 [file metabolites-12-01117-s001.zip › metabolites-1983203-supplementary.pdf]

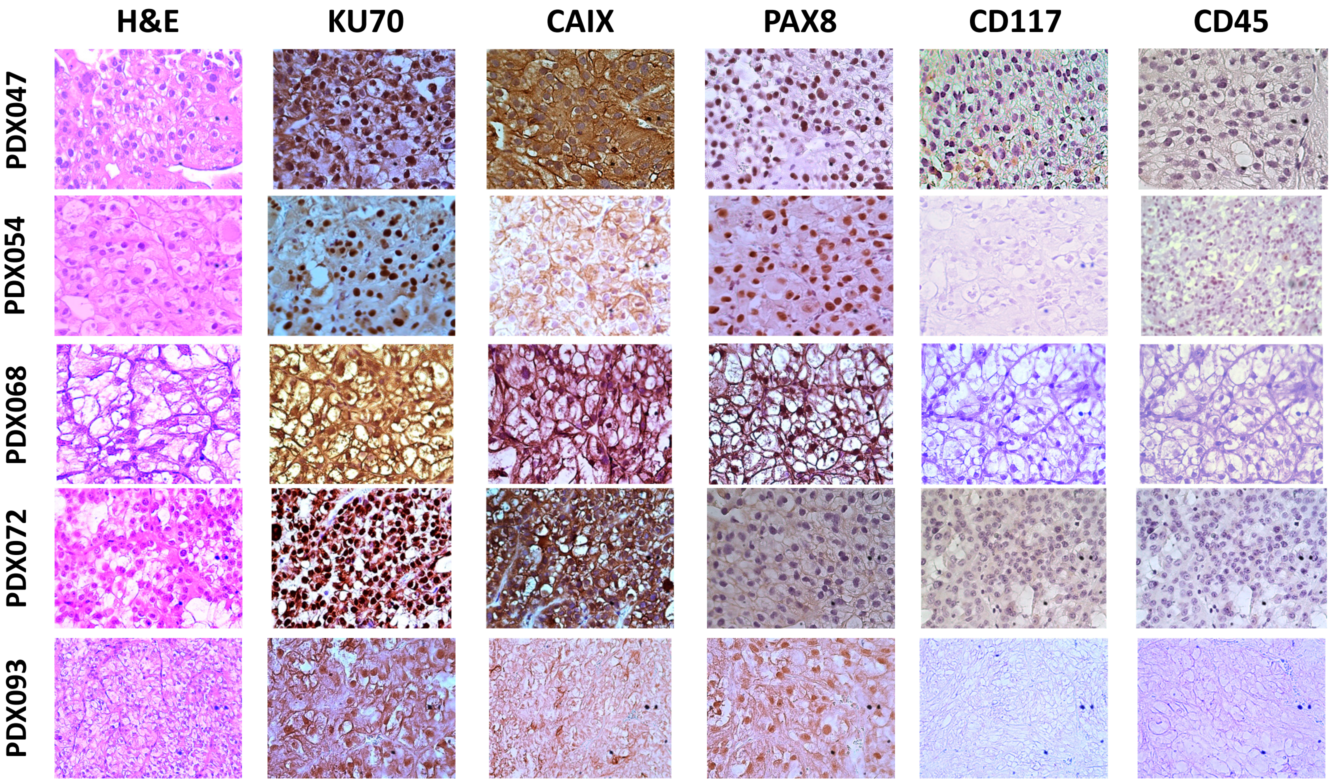

Figure S1. Expression of biomarkers by ccRCC PDX. Representative tumors from each PDX were stained with hematoxylin and eosin (H&E) and with antibodies Ku70 (specific for human cells), CAIX and PAX8 (expressed by ccRCC), CD117 (expressed by papillary RCC), and CD45 (expressed by lymphomas). Magnification X200.

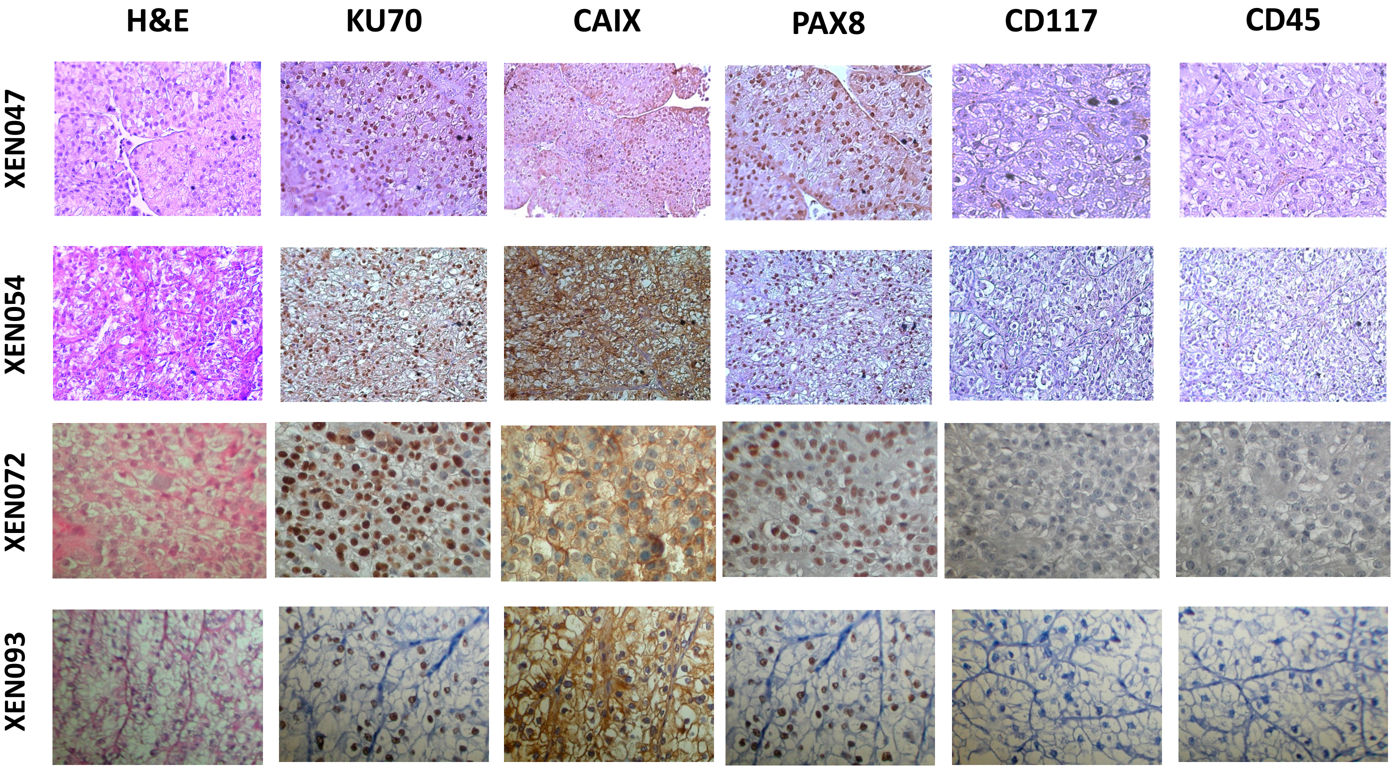

Figure S2. Expression of biomarkers by cell culture-derived XEN. Representative tumors from each XEN were stained with hematoxylin and eosin (H&E) and with antibodies Ku70 (specific for human cells), CAIX and PAX8 (expressed by ccRCC), CD117 (expressed by papillary RCC), and CD45 (expressed by lymphomas). Magnification X200.

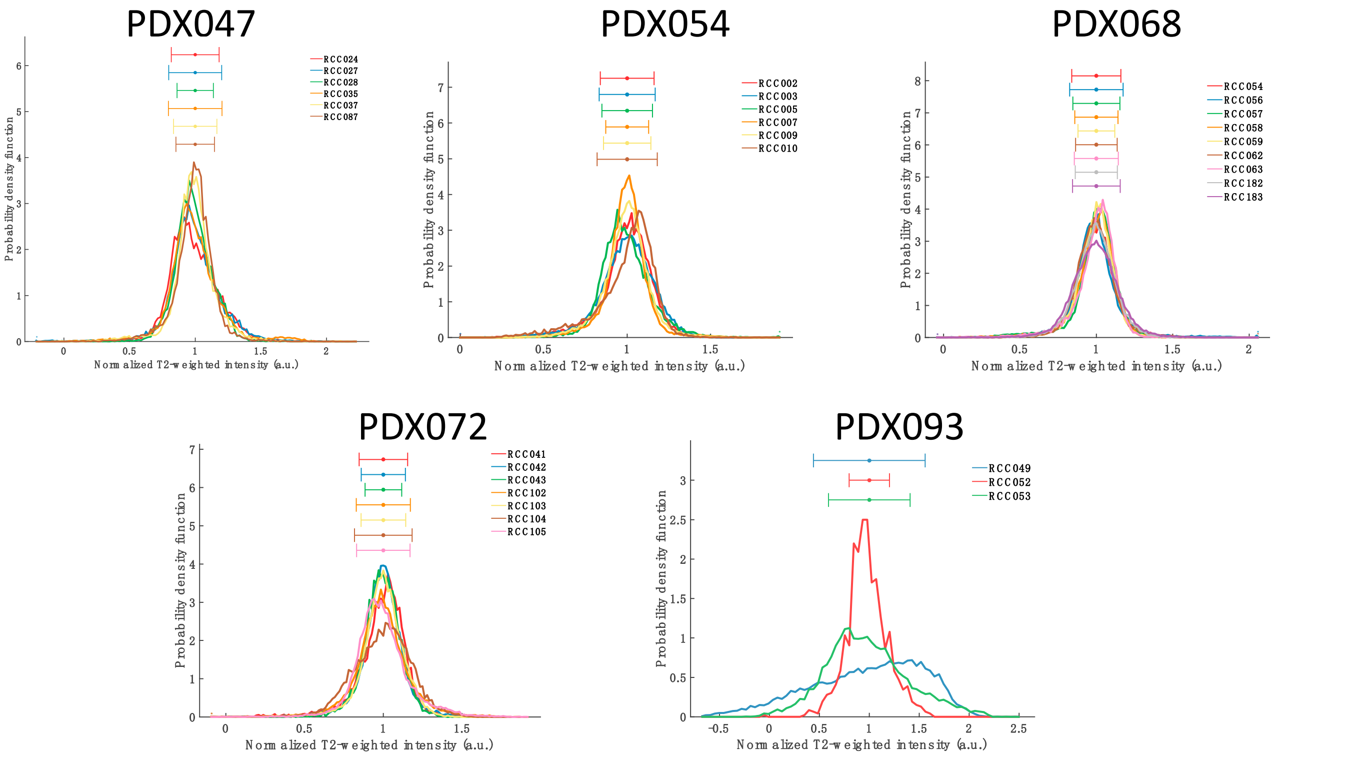

Figure S3. Histograms of normalized T2-weighted intensities of all tumors of each PDX (RCCxxx indicates tumor ID).

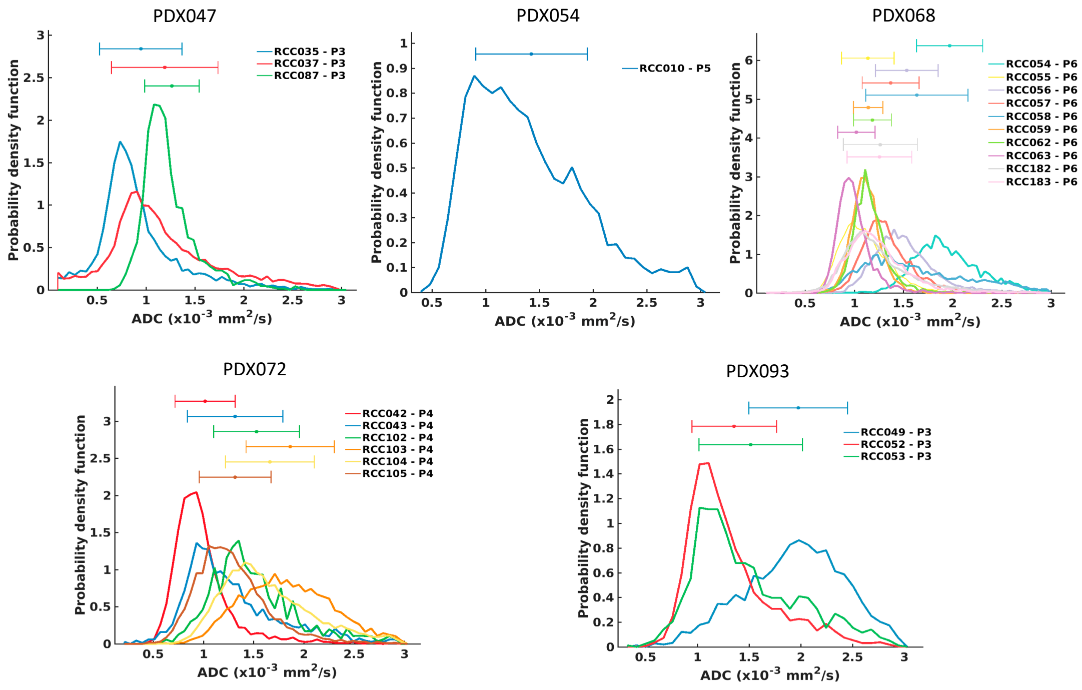

Figure S4. Histograms of ADC of all tumors of each PDX (RCCxxx indicates tumor ID and P is passage).

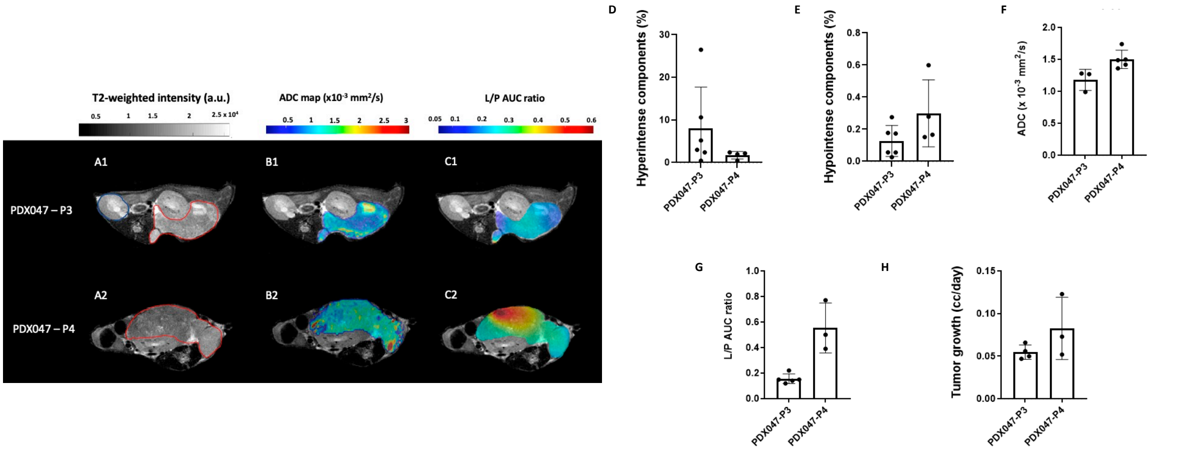

Figure S5. Representative T2-weighted MR images (**A1**-**2**), ADC maps (**B1**-**2**) and L/P AUC ratio (**C1**-**2**) for passage 3 (P3) and P4 of PDX047. (**D**) Hyperintense and (**E**) hypointense components (%) between P3 and P4 of PDX047 calculated from histograms of T2-weighted images. (**F**) Mean ADC of P3 and P4 in PDX047 showed significant water diffusion difference between passages. (**G**) The mean of the L/P AUC ratios for the two different passages of PDX047 also showed significant difference in glycolytic capacity. (**H**) The tumor growth rate (cc/day) was calculated as the slope of the last 3 tumor volumes prior to HP MRI and the time (in days) between the first and final measurement. The significance of the one-way ANOVA with a Tukey's multiple comparisons test is indicated above the plots. All $p < 0.05$ indicated statistical significance.

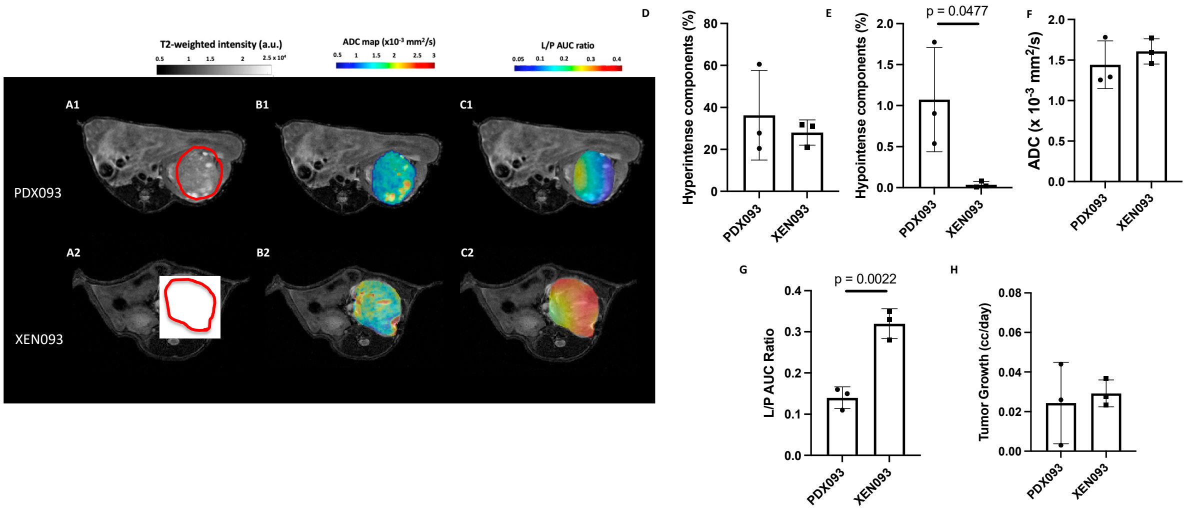

Figure S6**.** Comparison of features of PDX093 and XEN093. Panel on left: (**A1-2**) Representative T2-weighted MR images of PDX and XEN implanted under the murine renal capsule. Red lines were drawn along the borders of the tumors. (**B1-2**) Representative ADC maps overlaid on the T2-weighted images of PDX and XEN tumors. (**C1-2**) Representative L/P AUC ratio maps overlaid on the T2-weighted images of PDX and XEN tumors. A.u.= arbitrary units; AUC = area under the curve. Panels on the right: (**D**) the % hyperintense and (**E**) % hypointense components in all PDX and XEN tumors calculated from histograms of T2-weighted images; (**F**) ADC mean calculated for all PDX and XEN tumors; (**G**) the mean of the L/P AUC ratios for all PDX and XEN tumors; and (**H**) the tumor growth rate (cc/day). The significance of the one-way ANOVA with a Tukey's multiple comparisons test is indicated above the plots. All $p < 0.05$ indicated statistical significance.

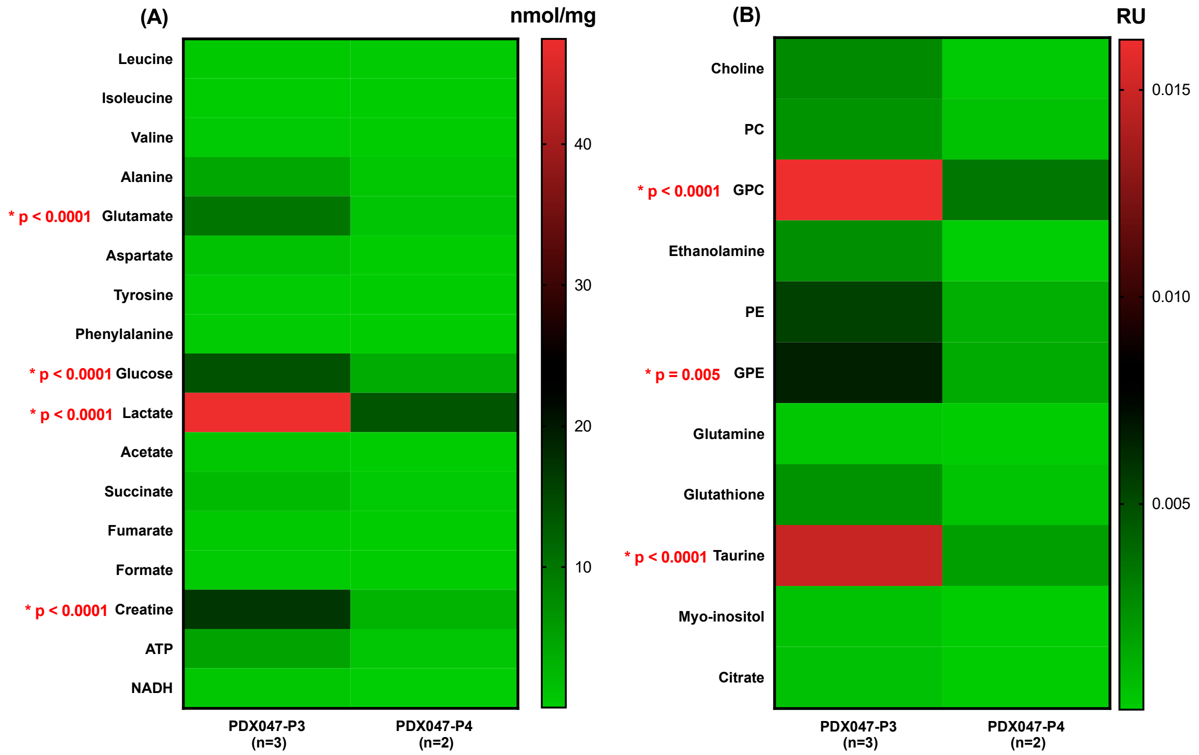

Figure S7. Heat maps showing (A) absolute concentrations and (B) relative concentration (RU) to TSP of steady state metabolite levels between passage 3 (P3) and passage 4 (P4) of PDX047. * denotes statistical significance

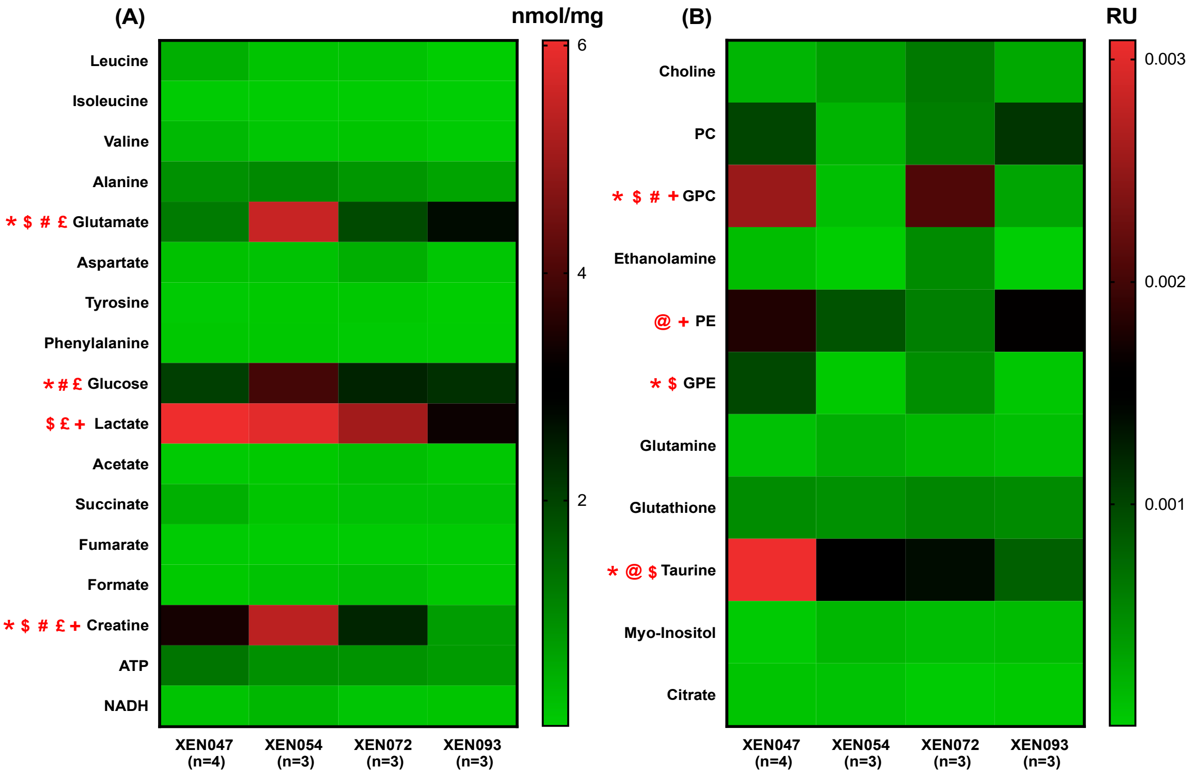

Figure S8. Heat maps of (A) absolute concentrations and (B) relative concentration (RU) to TSP of steady state metabolite levels among cell culture-derived xenografts (XEN). * - p<0.05, XEN047 and XEN054, @ - p<0.05, XEN047 and XEN072, $ - p<0.05, XEN047 and XEN093, # - p<0.05, XEN054 and XEN072, £ - p<0.05, XEN054 and XEN093, + - p<0.05, XEN072 and XEN093.

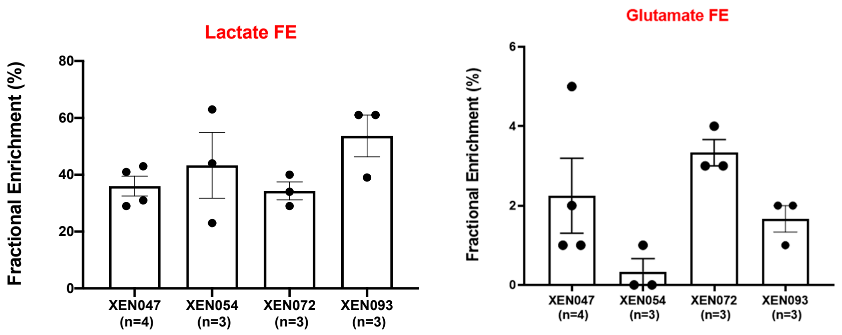

Figure S9. Comparison of fractional enrichment of lactate and glutamate among cell culture-derived xenografts (XEN).

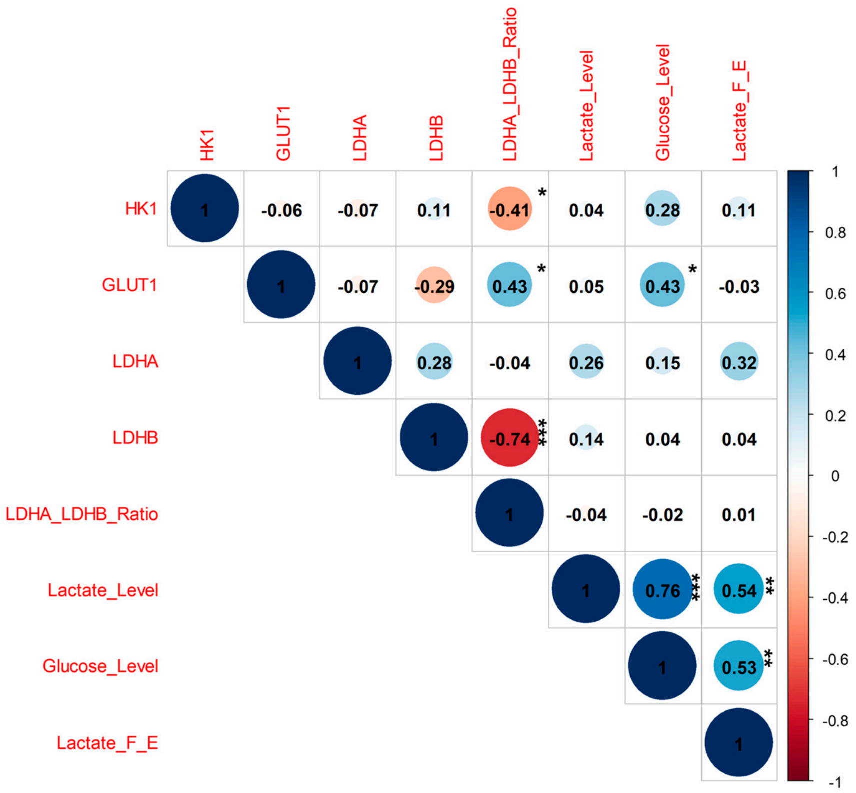

Figure S10. Correlation of metabolite and mRNA levels. * - p<0.05, ** - p<0.005, ***- p<0.0005

PDX047

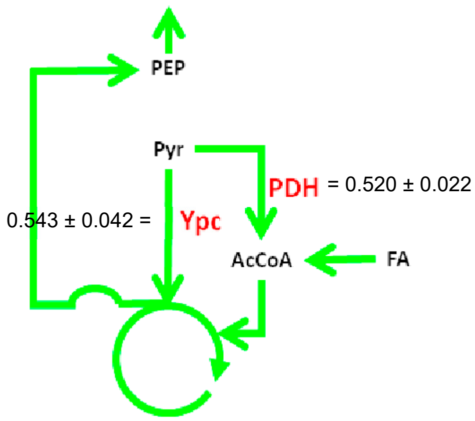

Figure S11. ^{13}C isotopomer modeling of [U-^{13}C] glucose-labeled PDX047 using tcaCALC software. PDH: pyruvate dehydrogenase; Ypc: pyruvate carboxylase

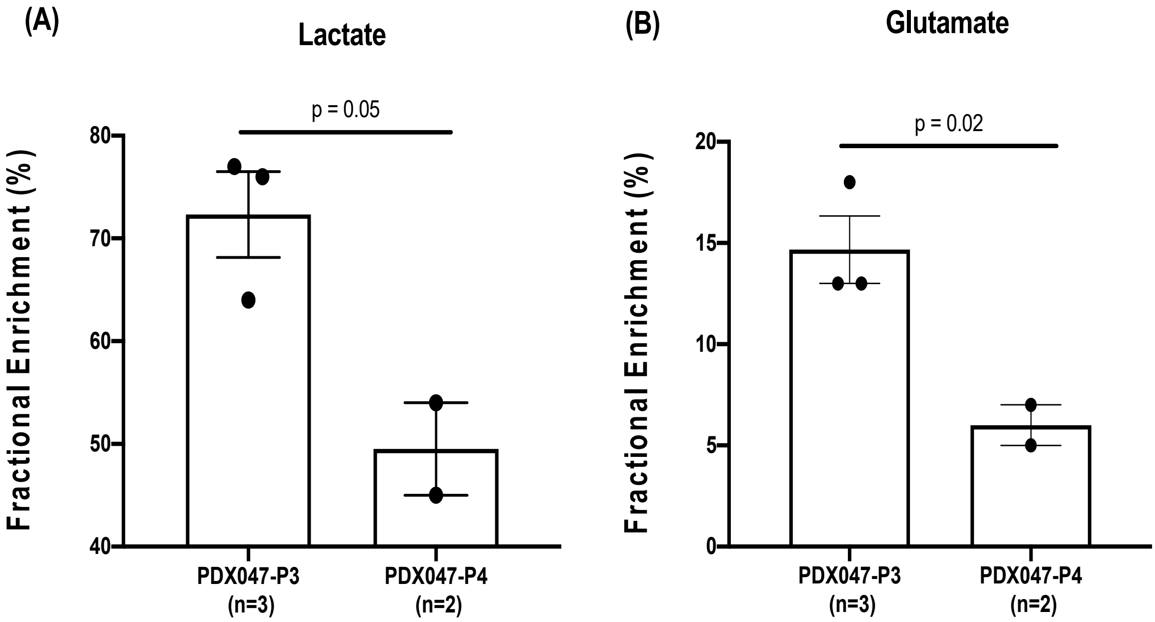

Figure S12. Comparison of fractional enrichment of (A) lactate and (B) glutamate between passage 3 (P3) and passage 4 (P4) of PDX047.

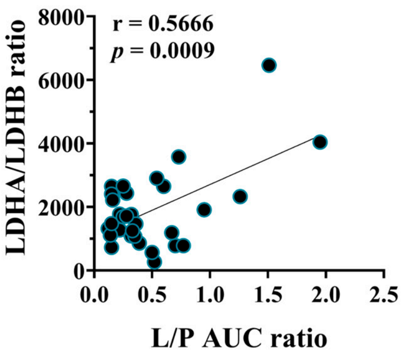

Figure S13. Correlation of LDHA/LDHB mRNA level to L/P AUC ratio.

Table S1. ^{1}H MR imaging parameters

Parameter	Axial T2-weighted Imaging	Axial Diffusion weighted imaging (b-values = 11, 108, 208, 308, 408, 508 and 1008 s/mm^2)
Pulse sequence	RARE (Rare factor = 8)	EPI (echo train = 8)
Echo time (ms)	7	30.5
Repetition time (ms)	100	2300
Flip Angle (◦)	40	90
Slice thickness (mm)	1	1
Slice gap (mm)	0	0
Matrix	192 x 192	96 x 96
Field of view (mm)	40 x 40	40 x 40
Averages	4	8
Acquisition time (s)	253.2	128.8

Table S2. Clinicopathologic characteristics of ccRCC specimens

	AGE	GENDER	FUHRMAN GRADE	STAGE	TISSUE	THERAPY
PDX047	72	Female	IV	TXNXM1	P[a]	Radiation and chemotherapy
PDX054	44	Male	IV	pT3aN0M1	M[b]	Chemotherapy
PDX068	74	Male	III	pT3aN0M1	P[a]	None
PDX072	65	Female	IV	TXNXM1	M[c]	Immunotherapy, radiation and chemotherapy
PDX093	73	Female	IV	pT3bNXM1	M[d]	None

[a]Primary tumor, obtained by radical nephrectomy; [b]metastasis to the colon; [c]metastasis to the lung, obtained at autopsy; [d]metastasis to the adrenal gland

Table S3. STR profiles of PDX and XEN

ID	PASSAGE	Loci								
		AMEL	CSF180	D13S317	D16S539	D21S11	D5S818	D7S820	TH01	TPOX	VWA
PDX047	P3	X	10	11	9	28	11,12	8,10	7,9	8	15,16
XEN047	P2	X	10	11	9	28	11,12	8,10	7,9	8	15,16
PDX054	P5	X	10,12	8,14	13,14	30,32.2	12,13	7,12	7,9.3	8,11	15,16
XEN054	P2	X	10,12	8,14	13,14	30,32.2	12,13	7,12	7,9.3	8,11	15,16
PDX068	P6	X,Y	11	12	11,13	27,32.2	11,13	8,9	7,9.3	8	17,18
PDX072	P4	X	10,11	12,13	12	28,29	10,11	9,11	6,9.3	8	16,17
XEN072	P2	X	10,11	12,13	12	28,29	10,11	9,11	6,9.3	8	16,17
PDX093	P3	X	12,14	12	11,13	28,31.2	10,12	12	8,9	8,11	14,17
XEN093	P2	X	12,14	12	11,13	28,31.2	10,12	12	8,9	8,11	14,17

Table S4. VHL genotype of PDX and XEN

ID	Generation	Mutation
PDX047	p3	c.503delC
XEN047	p2	c.503delC
PDX054	p5	c.492delG
XEN054	P2	c.492delG
PDX068	p6	c.470G>C
PDX072	p4	WT
XEN072	P2	WT
PDX093	p3	c.616insT
XEN093	p2	c.616insT
